# Hand grip strength as a physical biomarker of aging from the perspective of a Fibonacci mathematical modeling

**DOI:** 10.1186/s12877-018-0991-0

**Published:** 2018-11-29

**Authors:** Elena Ioana Iconaru, Manuela Mihaela Ciucurel, Luminita Georgescu, Constantin Ciucurel

**Affiliations:** 1 Department of Medical Assistance and Physical Therapy, University of Pitesti, Targul din Vale 1, 110040 Pitesti, Romania; 2 Department of Psychology and Communication Sciences, University of Pitesti, Pitesti, Romania; 3 Department of Natural Sciences, University of Pitesti, Pitesti, Romania

**Keywords:** Muscle strength, Hand dynamometry, Golden ratio, Decline, Older people

## Abstract

**Background:**

The Golden Ratio (GR) and the Fibonacci sequence have wide applications in biodiversity research, and recent studies indicate that the GR can be highlighted in the organization and physiological functioning of many body systems. The aim of this cross-sectional descriptive study is to determine the applicability of a mathematical model derived from the Fibonacci sequence to investigate the changes in hand grip strength (HGS) induced by the aging process.

**Methods:**

We assessed the HGS for both hands, using a Saehan hydraulic hand dynamometer in a group of autonomous elderly subjects. One hundred twenty 55-year-old subjects (58 males and 62 females) and seventy 89-year-old subjects (31 men and 39 women) were included in the study group. All subjects were completely independent or independent with minimal assistance in activities of daily living (ADL), as determined after applying the Barthel index of ADL. The data series were statistically processed using descriptive statistics (univariate analysis) and inferential statistical methods (the t test for unpaired groups, with effect size measure – Cohen’s d and the ratio of the means method).

**Results:**

The decline of the relative HGS between the two age groups can be expressed by values close to the GR value (*p* < 0.001), both in relation to body symmetry (left hand/right hand evaluation) and laterality (dominant hand/non-dominant hand evaluation), for both sexes. For the whole group of men and women, the rhythm of HGS decline may be expressed by a value (1.61) notably close to the GR, regardless of body symmetry or laterality.

**Conclusions:**

The common pattern of the relative HGS reduction between 55 and 89 years, as expressed by a value notably close to GR, can be considered to be an expression of a specific and predictable manifestation of the aging process, in the absence of disability.

## Background

### Applications of the Fibonacci series and Golden ratio in medicine

This article focuses on the study of the aging process from the perspective of mathematical modeling of certain physiometric indicator values, such as hand grip strength (HGS). We propose this approach to achieve possible interpretation schemes for the prognosis of the aging process among the population, seeking several practical applications of the Fibonacci mathematical modeling.

The Fibonacci sequence represents a string of natural numbers (1, 1, 2, 3, 5, 8, 13, 21, 34, 55, 89, 144, 233 …), with each number being the sum of the two preceding ones. At the same time, the ratio between any given number in a Fibonacci sequence and its predecessor tends towards an irrational number (1.618….), known as the “Golden Ratio – (GR)”, phi or φ among the Greek mathematicians [1].

The Fibonacci series and Golden Ratio has been the subject of study for numerous researchers investigating the existence in the environment of certain repetitive representations derived from this mathematical model, known as fractals [2–4]. These repetitive representations underline the occurrence of the phyllotaxis phenomena, which is strongly related to the Fibonacci sequence [2]. At microscopic and macroscopic levels, the repetitions of fractals can be seen in various biological areas [3, 4].

In medicine and related fields, the use of the models derived from the Fibonacci series seems to be even more surprising and attractive. An area with great applicability of fractal geometry is that of imagery, such as MRI and CT examinations of the brain or other organs [5–7]. Studies have reported the existence of the GR in cardiovascular hemodynamic parameters, both in normal and pathological contexts [8–14].

There are other notable examples to demonstrate the GR utility in human pathology. For example, GR can be used as a basic mathematical benchmark for assessing hand and heart proportions for optimal embryologic development [15]. However, for the inferential generalization of results and the extension of such research from the field of physiology towards pathology, more comprehensive studies would be required [16].

### Applications of the Fibonacci series and Golden ratio in the human hand and new perspectives of mathematical modeling

Golden Ratio was also found in the morphology of the hand and forearm. Additionally, numerous studies suggest that human limb bone lengths and volumes follow certain mathematical models of expression [17–20]. Thus, according to several authors, the ratio between the lengths of the forearm and the hand is approximately 1.618 [18], and the ratio of forearm plus hand to forearm is close to the value of the GR [19]. Similarly, the ratios between the lengths of the metacarpals and the corresponding phalanges are close to the phi value [20].

A recent study has also presented evidence of the presence of the golden curve in length proportions of human upper limbs, from a functional perspective [21]. The already classic studies of Littler [22] identified the hand’s grip adaptability as being determined by the specific anatomical shape of the skeletal arc of the hand. Thus, the curves made during hand movements are conditioned by the existence of GR value ratio between the lengths of carpometacarpal and phalangeal bones.

Also, the interarticular distances of the fingers follow the Fibonacci sequence and the motion of the tips of the fingers, during flexion and extension, follow an equiangular spiral, derived from the same mathematical model [22–24]. However, there are still controversies and differing interpretations of the anatomic and clinical data in terms of applicability of the Fibonacci series and GR at the level of the hand structure and functionality [25–27].

The GR and Fibonacci sequence has been the subject of multiple theoretical and practical approaches in history, and recent research extends applicability of the GR and Fibonacci sequence from the field of the exact sciences towards biological and biomedical sciences. In fact, the latest perspective on this domain refers to the development of nanotechnologies, such as the research on quasicrystals and on the phenomena of nanoscale symmetry hidden in solid-state matter [28].

Finally, the design and analysis of bipedal humanoid robots can be achieved by inferential application of such mathematical models described in Mechatronics Engineering [29]. Similarly, certain studies have reported the applicability of GR at the level of the phases of bipedal gait, specific to humans, under normal and pathological conditions [30, 31].

### Hand grip strength as a physical biomarker of aging and/or morbidity

All of the aspects mentioned above become more exciting if we consider the ontogenetic perspective of human development. Therefore, the identification of certain mathematical models to quantify the aging process of specific structures of the human body could provide arguments for a more accurate prediction of the consecutive functional decline.

The aging of the human body is best characterized by the progressive regression of the homeostatic resources, regardless of the hierarchical level of organization to which we refer to (cells, tissues, organs, body systems). In this context, the use of the physical capacities as an indicator of autonomy decline in the elderly is imposed with necessity in order to delimit more accurately the normal process of aging from the pathological one.

Evaluation of HGS can serve as an indicator for development and coordination, and can facilitate the diagnosis of neurological disorders related to motor learning and perception. The same procedure can also be applied in various other morbidity contexts.

HGS is considered a reliable measure to assess overall muscle strength and it is used in clinical settings for the diagnosis of sarcopenia and frailty across the lifespan [32]. In brief, the decreasing muscle quality is associated with the age-related decline in HGS in middle-aged and older adults [33]. Neural factors also contribute, in part, to decreased HGS during aging. Thus, the age-related morpho-functional impairments of the human nervous system, which affect the muscle performance, refer to the changes in spinal and supraspinal properties and the alteration in neurotransmitters [34, 35]. Hand grip strength is a significant predictor and biomarker of health, morbidity, disability, and mortality, not only in the elderly, but also in middle-aged and young people [36].

In the elderly, HGS has been proposed as an indicator of health, vitality, illness or disability, and the rate of aging indirectly influences the hand’s function as an organ [37, 38]. The study of the HGS in the elderly represents an exciting approach, and different researchers over time have demonstrated the significant correlation of this parameter with various morpho-functional, social, and psychometric features [39]. In several studies, the interpretation focused on the multisystemic deficiencies that would negatively affect the biomechanical parameters of the hand (sarcopenia in particular), especially for patients with chronic diseases and multimorbidity [40].

In the context of aging and/or geriatric pathology, the reduction of HGS has a negative influence on elderly quality of life, which is easy to understand if we analyze the role of the hand in daily occupational activities. There are many recent studies showing that HGS is correlated with multiple morbid conditions, including the risk of developing future disability or premature death, particularly in the context of the age-related sarcopenia [41]. However, caution is needed in extrapolating results, while considering a broader context and nevertheless placing the obtained values in a dynamic frame [42].

## Methods

### Aim of the study and premise

The practical objective of this cross-sectional descriptive study focused on determining the applicability of a mathematical model derived from the Fibonacci sequence to investigate the changes in HGS induced by the aging process.

The hand grip is determined by the anatomical structures of the hand and forearm, and these anatomical structures have been the subject of prior studies that confirmed various applications of the previously mentioned mathematical modeling. The Fibonacci numbers overlapping with human ages are 1, 2, 3, 5, 8, 13, 21, 34, 55, 89; the next value is 144, which is out of human age range.

From the ontogenetic perspective, the age of 55 years represents the threshold that is considered the entrance into the older adult or young-old stage [43, 44]. In addition, the age of 89 years is perceived as the upper limit of the first oldest-old subgroup (85–89 years, respectively). For this population segment, there are multiple related references, such as the Leiden-85 plus study [45]. Furthermore, in terms of disability onset, the homogeneity of the elderly in the over 85-year-old group is considerably different from those at the age of 90 years [46]. Therefore, the age of 89 years can be considered a critical time (cut-off) to investigate the relationship with hand functionality [47].

Given the above data, we can formulate, as a working hypothesis, that it is possible to identify a mathematical relationship between the values of the HGS for the two age categories (55 and 89 years old), eventually considering the variables related to the level of the autonomy of the subjects and the anthropometric variables. Given that the HGS was extensively studied as a predictive indicator of health/disability and longevity/mortality, we performed a cross-sectional descriptive study with the potential to generate predictive models of the aging rhythm from the perspective of the GR.

### Participants

We used a group of 190 autonomous elderly subjects, selected from the general population of four Romanian counties, by accessing the regional social network of support for the elderly. One hundred twenty 55-year-old subjects (58 males and 62 females) and seventy 89-year-old subjects (31 men and 39 women) were included in the study group.

For each participant, we obtained consent to be involved in the study in accordance with the ethics of research on human subjects. All subjects were completely independent or independent with minimal assistance in activities of daily living (ADL), as determined by applying the Barthel index of ADL [48]. This criterion was applied by selecting subjects with a score of 85–100 points upon administration of the mentioned tool [49, 50]. When selecting subjects, we also excluded individuals with various forms of acute pathology of the upper limb, recent injuries, arthritis, and neuromuscular disorders (*n* = 28).

### Data acquisition

The study was conducted over 6 months in 2017. Through anamnesis, we collected data from each participant, especially regarding the previous medical history. Next, for each of them, we assessed the HGS for both hands using a Saehan model hydraulic hand dynamometer (MSD Europe bvba, Begium). The results were recorded in kilograms force (kgf). An important aspect is the measuring range of the device, which is between 0 and 90 kgf (or 200 lbf). The device’s handle allows fine adjustment in 5 steps, ranging 3.8 cm to 8.9 cm, in order to fit individual’s palm size. The instrument has good validity and reliability references for clinical use, similar to the hydraulic Jamar dynamometer [51].

As a standard procedure, participants sat with their elbow flexed at 90°, the dynamometer’s base was set on the first metacarpal bone (inside the palm), and the handle was placed in contact with half of the last four fingers. To this end, the dynamometer’s handle was adjusted for each subject in accordance with constitutional parameters. When the subject was ready, he began to strain the device in a maximal isometric effort with a duration of 3–5 s. No other body movement was allowed during testing. The subject was encouraged to maintain high concentration, as this was a required condition for maximal results.

The test was repeated three times for each hand after a recovery period of 20–30 s for each measurement. The best result for each hand was taken into consideration, also noting the dominant hand for each subject. Thus, the dominant hand was indicated as the one preferred for ADL.

Simple anthropometric measurements were also performed according to standard procedures for determining body height (H) and body mass (M). H was measured with a portable stadiometer (Seca 213, Seca GmbH & Co. Kg, Hamburg, Germany) and M with a portable digital scale (Omron HN-286, Omron Corporation, Kyoto, Japan). All measurements were realized in the morning, between 8 and10 a.m., with participants in a standing position. We chose these anthropometric indicators as they are the most commonly used in correlational studies for HGS [52].

### Outcomes and statistical analysis

Using H and M values we calculated the body mass index (BMI, measured in kg/m^2^) for each subject in accordance with the formula in which H is expressed in m and M in kg:

$$ \mathrm{BMI}=\frac{M}{H^2} $$

HGS data were initially grouped by sex for the left and right hand separately. Next, we also grouped data by sex, for the dominant hand and non-dominant hand separately. We considered this important because many studies insist on the interference of laterality in interpreting the values of HGS [53].

The last parameter determined for each subject was the relative HGS, which is defined as the ratio between the summation of HGS for both hands and BMI. This synthetic indicator is highly appreciated in correlational studies related to HGS because it allows the possibility to interpret the results within the anthropometric context [54]. Therefore, these data were grouped in accordance with the algorithm mentioned previously.

In most cases, normative data for HGS are divided into age and gender subgroups and all studies show average higher values of HGS in men at all ages [55, 56]. Sex is the strongest multivariate term that explains the variance of grip strength, and for this reason, HGS data are usually stratified by sex [57].

The data series were statistically processed using descriptive statistics (univariate analyses), by determining means, weighted means and standard deviations (SD). Next, we applied inferential statistical methods in order to evaluate the differences between means by using the t test for unpaired groups, the magnitude of the difference between groups (Cohen’s d effect size measures) and the ratio of the means method.

The ratio of the means has been of interest in the classical inference from infinite populations for the construction of unbiased ratio estimators [58]. For example, in biometric studies, this composite indicator is useful as a consistent parameter [59], because it conveys the overall effect on the population as a whole by combining data from different subgroups [60].

## Results

The statistical indicators for the data series are shown in Tables 1 and 2.

**Table 1:** Statistical indicators for the subgroups of men

	Men, 55 years old *n* = 58		Men, 89 years old *n* = 31		Statistical differences between means	
Variable	Mean	SD	Mean	SD	t	p
M (kg)	80.50	11.14	65.84	9.05	–	–
H (cm)	173.38	7.42	167.81	7.81	–	–
BMI (kg/m^2^)	26.73	2.77	23.36	2.68	–	–
R HGS (kgf)	45.92	3.02	26.19	2.42	31.35	< 0.001
L HGS (kgf)	40.80	2.88	22.15	3.12	28.28	< 0.001
Relative HGS – R/L (kgf/kg/m^2^)	3.27	0.27	2.08	0.19	21.75	< 0.001
D HGS (kgf)	46.64	2.42	26.50	2.39	37.55	< 0.001
ND HGS (kgf)	40.08	1.72	21.84	2.66	39.16	< 0.001
Relative HGS – D/ND (kgf/kg/m^2^)	3.27	0.27	2.08	0.19	21.75	< 0.001

*M* body mass, *H* body height, *BMI* body mass index, *HGS* hand grip strength, I right, *L* left, *D* dominant, *ND* non-dominant, *SD* standard deviation, *n* number of subjects, *t* t test value, *p* threshold of statistical significance

**Table 2:** Statistical indicators for the subgroups of women

	Women, 55 years old *n* = 62		Women, 89 years old *n* = 39		Statistical differences between means	
Variable	Mean	SD	Mean	SD	t	p
M (kg)	65.98	7.72	56.59	6.80	–	–
H (cm)	159.98	6.57	156.18	4.70	–	–
BMI (kg/m^2^)	25.76	2.43	23.18	2.43	–	–
R HGS (kgf)	30.25	2.02	16.63	1.79	34.42	< 0.001
L HGS (kgf)	28.58	1.49	15.60	1.39	43.68	< 0.001
Relative HGS – R/L (kgf/kg/m^2^)	2.30	0.17	1.40	0.14	27.89	< 0.001
D HGS (kgf)	30.44	1.92	16.79	1.66	36.62	< 0.001
ND HGS (kgf)	28.38	1.37	15.43	1.40	45.76	< 0.001
Relative HGS – D/ND (kgf/kg/m^2^)	2.30	0.17	1.40	0.14	27.89	< 0.001

*M* body mass, *H* body height, *BMI* body mass index, *HGS* hand grip strength, *R* right, *L* left, *D* dominant, *ND* non-dominant, *SD* standard deviation, *n* number of subjects, *t* t test value, *p* threshold of statistical significance

As it can be observed in Tables 1 and 2, all of the differences HGS value means between the investigated subgroups were statistically significant (*p* < 0.001). In addition, the magnitude of the differences between subgroup means corresponded to a large effect size, in all situations (Cohen’s d > 0.80, Table 3).

**Table 3:** The mean difference and the Cohen’s d effect size from the inferential analysis

Subgroups	Mean difference	Cohen’s d
R HGS, men 55 years old (*n* = 58) versus R HGS, men 89 years old (*n* = 31)	19.73	6.98
L HGS, men 55 years old (*n* = 58) versus L HGS, men 89 years old (*n* = 31)	18.65	6.29
Relative HGS – R/L, men 55 years old (*n* = 58) versus relative HGS – R/L, men 89 years old (*n* = 31)	1.19	4.85
D HGS, men 55 years old (*n* = 58) versus D HGS, men 89 years old (*n* = 31)	20.14	8.36
ND HGS, men 55 years old (*n* = 58) versus ND HGS, men 89 years (*n* = 31)	18.24	8.72
Relative HGS – D/ND, men 55 years old (*n* = 58) versus relative HGS – D/ND, men 89 years old (*n* = 31)	1.19	4.85
R HGS, women 55 years old (*n* = 62) versus R HGS, women 89 years old (*n* = 39)	13.62	7.04
L HGS, women 55 years old (*n* = 62) versus L HGS, women 89 years old (*n* = 39)	12.98	8.94
Relative HGS – R/L, women 55 years old (*n* = 62) versus relative HGS – R/L, women 89 years old (*n* = 39)	0.9	5.66
D HGS, women 55 years old (*n* = 62) versus D HGS, women 89 years old (*n* = 39)	13.65	7.48
ND HGS, women 55 years old (*n* = 62) versus ND HGS, women 89 years old (*n* = 39)	12.95	9.37
Relative HGS – D/ND, women 55 years old (*n* = 62) versus relative HGS – D/ND, women 89 years old (*n* = 39)	0.9	5.66

*HGS* hand grip strength, *R*: right *L* left, *D* dominant, *ND* non-dominant, *n* number of subjects

It is important to mention that the dominant hand was the right hand for 89.7% of the 55-year-old men (*n* = 52), 88.7% of the 55-year-old women (*n* = 55), 87.1% of the 89-year-old men (*n* = 27) and 87.2% of the 89-year-old women (*n* = 34).

Next, we considered it appropriate to report the means of the HGS values between subgroups in order to estimate the age-induced functional decline in HGS, between the two selected ages (55 years and 89 years). By calculating a ratio of mean values, which has no specific units of measurement, we can transform the analyses of continuous variables (such as HGS) into a simplified clinical interpretation of a process (such as aging).

**Fig. 1:**
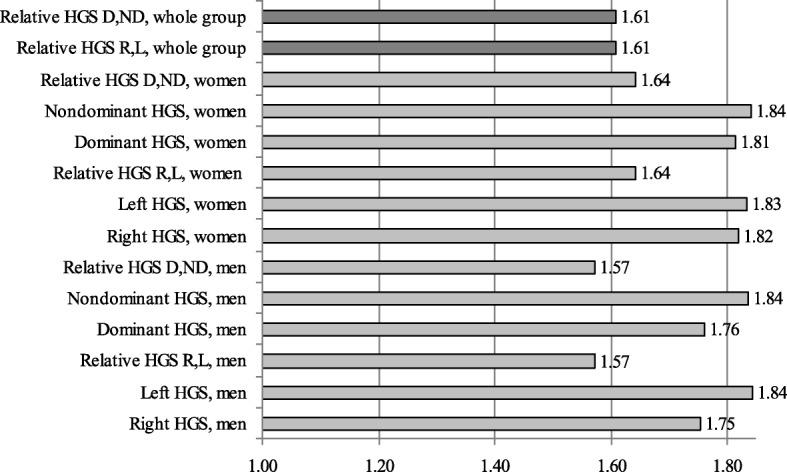
The ratios between HGS means for the investigated subgroups. Note: *HGS* hand grip strength, *R* right, *L* left, *D* dominant, *ND* non-dominant

From Fig. 1, it can be observed that this decline is relatively uniform for both men and women, regarding body symmetry (right hand/left hand) or laterality (dominant hand/non-dominant hand). The values closest to the GR value (1.61) correspond to the situation of reporting the relative HGS values at the level of the whole group, when taking into account the body symmetry (left hand/right hand evaluation) and the laterality (dominant hand/non-dominant hand evaluation). The two investigated ages (55 and 89 years), considered from the Fibonacci sequence, are thus important from an ontogenetic perspective, regarding the rhythm of decline of HGS.

## Discussion

HGS suffers a significant decline induced by aging, which can be quantified using a process of mathematical modeling. Thus, for this analysis, we consider two ages that overlap with consecutive numbers from the Fibonacci series (55 years and 89 years) as reference values. In particular, the decline of the relative HGS between the mentioned ages can be expressed by values close to the GR value, both in relation to body symmetry (left hand/right hand evaluation) and laterality (dominant hand/non-dominant hand evaluation), for both sexes: for men, the relative HGS decreases 1.57-fold, and for women, it decreases 1.64-fold.

When using the relative HGS ratios, indicators that adjust the HGS values according to an important anthropometric parameter (BMI), for the whole group of men and women, as weighted means of the ratios for the entire study group, the rhythm of decline may be expressed by a value notably close to the GR (a decrease of 1.61-fold), regardless of body symmetry or laterality.

The innovation of this research lies in the simultaneous approach of the evaluation of the HGS of both hands with reference to body symmetry (right hand/left hand), and laterality (dominant hand/non-dominant hand); most other authors focused on only one perspective in reporting their results. In addition, the use of the relative HGS for the overall assessment of subjects is advantageous because it offers a formula that sums the HGS values for the right hand and the left hand, and the dominant hand and the non-dominant hand, respectively. In these circumstances, the effect of body symmetry or laterality disappears.

Hand grip impairment in the elderly is closely related to the multisystemic deterioration that is responsible for the decline of neuromotor performances. There is abundant clinical evidence that clearly indicates that HGS has been affected in the elderly [39, 56]. The global loss of manual function can be quantified through a clinical test, during senescence. Based on these results, the origin of particular deficits identified can be more precisely localized in order to propose therapeutic intervention strategies with relevance to rehabilitation in different clinical contexts.

Mathematical modeling of HGS values can serve to identify the rhythm of normal aging. In this regard, the two age groups, aged 55 years and 89 years, have been investigated as possible landmarks in our study. Other authors have attempted to describe human development according to the Fibonacci sequence. Using mathematical modeling, Waskom identified a natural progression of human ontogenesis with eight life stages. According to this model, the interval between 55 and 89 years represents the elderly stage, characterized by progressive physical decline [61, 62]. After progressing through the adult stage and after reaching the critical point of 55 years of age (older adulthood), a decrease in physical strength and stamina is progressively observed, with inter-individual variations, and the older adult begins to become a potential beneficiary of social services [63].

In practical terms, each of these predetermined stages of human development follows an optimal epigenetic biological pattern. This phenomenon can be explained starting from the occurrence of Fibonacci sequence in biological cell division and self-organizing and dynamic systems, as a premise for the logarithmic normal body growth, based on GR [58]. Identification of the ontogenetic transitional phases may be useful in terms of designing more effective prophylactic or therapeutic interventions for specific individuals who are facing major and critical life-changing events [64].

The obtained mean HGS values are comparable with the extracted data from various nomograms corresponding to elderly stages, eventually detailed through decile or centile analysis [56, 65, 66]. However, most studies focus on determining particular mean values by age group (usually five years intervals). Therefore, predictions can be done on certain reference values for age point values only by using regression equations [57, 67].

Additionally, various authors have found differences in terms of HGS values in certain population groups. These reported differences are even higher when other composite variables are taken into account, such as elderly morbidity, ethnicity, and hereditary factors [68].

In our case, we were less interested in strictly interpreting the HGS mean values for the two ages than in the dynamics of this indicator. In brief, the rhythm of HGS functional decline correlated with the anthropometric profile can be explained by the process of senescence, per se, in the investigated group. Clearly, the absence of disability among subjects ensured the exclusion of variables that interfere with physiological aging.

From the analysis of the results obtained through mathematical modeling, we are able to suggest that the ratio between the mean relative HGS for 55-year-old subjects and the mean relative HGS for the 89-year-old subjects can be expressed by a value notably close to GR.

The results of the study open other possibilities of future investigations, such as the extension of the proposed mathematical modeling in various morbidity contexts, for the prediction of longevity, disability or mortality and for the population screenings. Another possible research implication would be to examine how anthropometric factors (particularly, digit length and related hand characteristics) may contribute to grip strength. Therefore, we could extend further the idea of applying the GR to the structure and functionality of the hand. The evaluation of HGS in other age groups that fit in the Fibonacci sequence could generate new interesting research hypothesis. In practical terms, by validating of certain rhythms of decline of HGS between multiple ages, and in different clinical contexts, which follow a mathematical model, we could better quantify the rate of normal aging versus pathological aging.

## Limitations of the study

As a descriptive study, our findings cannot establish causal relationships between the recorded parameters. Also, this research did not focus on assessing the variability of HGS induced by individual elderly morbidity (inherent especially for the 89-year-old subjects), which was relatively diverse at the study group level, but did not generate disability.

In interpreting the evolution of certain functional parameters, a prudent approach would be beneficial in the context of preserving the rigorous scientific methodology. Thus, at interpretative level, we have to overcome the tendency to exhaustively apply certain patterns that seem to be generally valid, regardless of context. In this case, the most appropriate attitude is one of caution against what Bennett called “the tyranny of the golden mean” [66]. In practical terms, inter-individual differences in investigated variables with intrinsic significance can be mitigated through the process of generalization in the context of using the central tendency indicators [69, 70].

## Conclusions

Starting with the already proven classical applications of Fibonacci series and GR in the hand morphology and functionality, we succeeded in highlighting a new version of mathematical modeling that can demonstrate the HGS decline during aging. The common pattern of diminishing relative HGS between 55 and 89 years, expressed by a value notably close to GR, can be considered an expression of a specific and predictable manifestation of the aging process in the absence of disability. These new data suggest that the human aging process could follow highly repeatable mathematical patterns, and the recorded results can provide research starting points in the field of gerontology and geriatrics.

## Abbreviations

ADL: Activities of daily living
BMI: Body mass index
D: Dominant
GR: Golden Ratio
H: Body height
HGS: Hand grip strength
L: Left
M: Body mass
n: Number of subjects
ND: Non-dominant
R: Right
SD: Standard deviation
